# Past, present, and future of sustainable intensive care: narrative review and a large hospital system experience

**DOI:** 10.1186/s13054-024-04937-9

**Published:** 2024-05-09

**Authors:** Faisal N. Masud, Farzan Sasangohar, Iqbal Ratnani, Sahar Fatima, Marco Antonio Hernandez, Teal Riley, Jason Fischer, Atiya Dhala, Megan E. Gooch, Konya Keeling-Johnson, Jukrin Moon, Jean-Louis Vincent

**Affiliations:** 1 Center for Critical Care, Houston Methodist, 6550 Fannin St., Houston, TX 77030 USA; 2 Office of Sustainability, Houston Methodist, 6550 Fannin St., Houston, TX 77030 USA; 3 Department of Surgery, Houston Methodist, 6550 Fannin St., Houston, TX 77030 USA; 4 Center for Health Data Science and Analytics, Houston Methodist, 6550 Fannin St., Houston, TX 77030 USA; 5 Erasme University Hospital, Université Libre de Bruxelles, Brussels, Belgium

**Keywords:** Critical care, Environmentalism, Environmental policy

## Abstract

Healthcare systems are large contributors to global emissions, and intensive care units (ICUs) are a complex and resource-intensive component of these systems. Recent global movements in sustainability initiatives, led mostly by Europe and Oceania, have tried to mitigate ICUs’ notable environmental impact with varying success. However, there exists a significant gap in the U.S. knowledge and published literature related to sustainability in the ICU. After a narrative review of the literature and related industry standards, we share our experience with a Green ICU initiative at a large hospital system in Texas. Our process has led to a 3-step pathway to inform similar initiatives for sustainable (green) critical care. This pathway involves (1) establishing a baseline by quantifying the status quo carbon footprint of the affected ICU as well as the cumulative footprint of all the ICUs in the healthcare system; (2) forming alliances and partnerships to target each major source of these pollutants and implement specific intervention programs that reduce the ICU-related greenhouse gas emissions and solid waste; and (3) finally to implement a systemwide Green ICU which requires the creation of multiple parallel pathways that marshal the resources at the grass-roots level to engage the ICU staff and institutionalize a mindset that recognizes and respects the impact of ICU functions on our environment. It is expected that such a systems-based multi-stakeholder approach would pave the way for improved sustainability in critical care.

**Graphical abstract:**

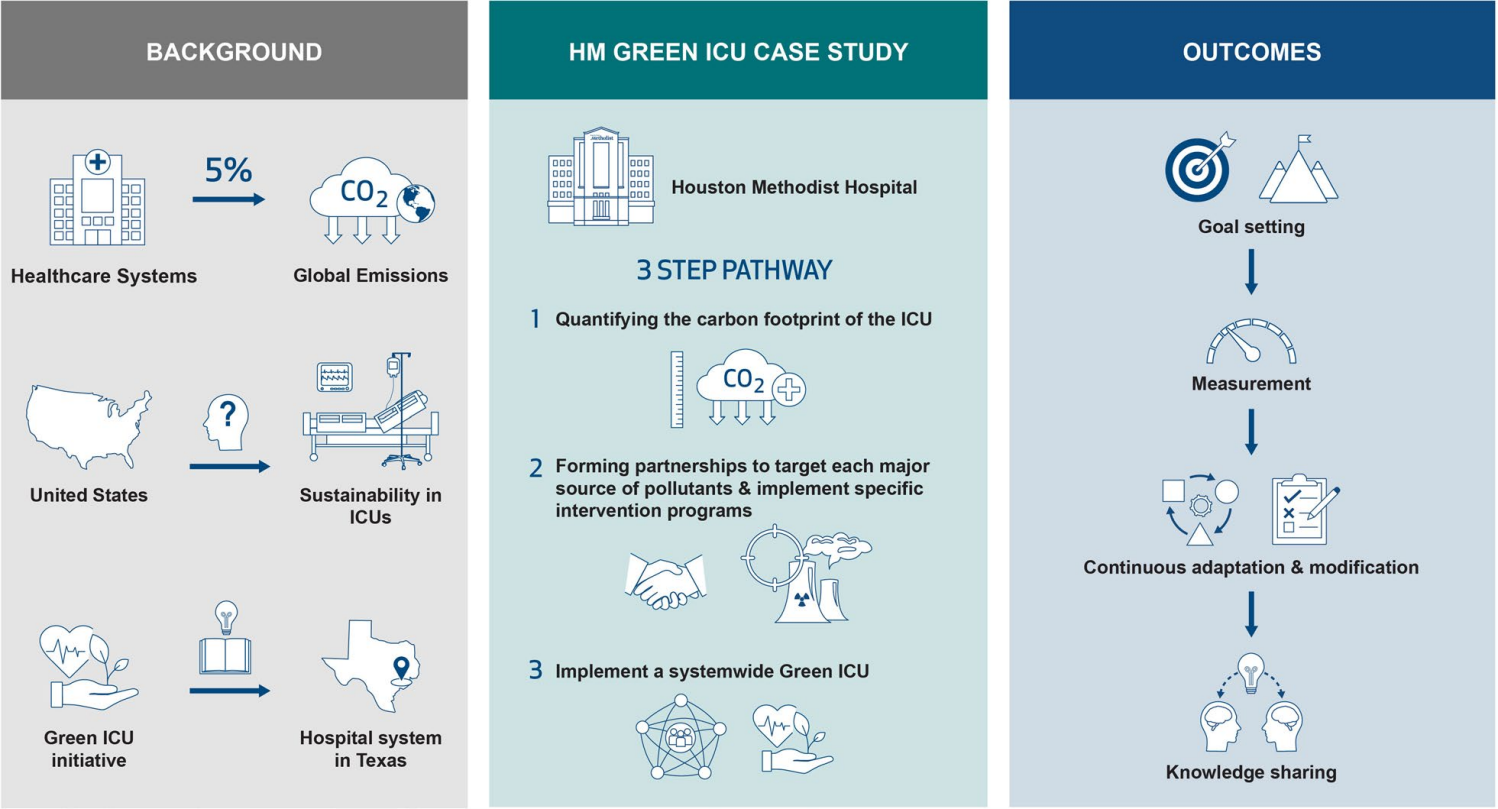

## Introduction

Climate change is one of the major grand challenges humanity faces in the twenty-first century. The burning of fossil fuels is the largest contributor to climate change, responsible for over 75% of greenhouse gas (GHG) emissions [1]. Among many industries that contribute to GHG emissions, the complexity and growth of global healthcare systems have led to an exponential impact from an environmental standpoint. Healthcare’s carbon emissions and footprints are estimated to be as high as 5% globally, with the United States taking the plurality (one-quarter) of this share [2, 3]. Nations with overall high footprints attribute a substantial percentage coming from healthcare (e.g., an Australian assessment found 7% of total carbon was attributable to healthcare industry [4]); even when the overall industry-specific footprint is lower (as in China), proportions are concentrated around medical supply chain [5]. The movement to plastic and disposable products has also been a key facilitator to a rapid expansion of care, albeit at the significant cost to the environment [6]. Other healthcare-related environmental factors include carbon emissions in procurement wastes [7], direct energy consumptions of equipment [8], and travel options for patients [9].

Huffling and Schenk [10] described a vicious cycle between healthcare and climate change: the healthcare sector’s negative impact on environmental harm contributes to illness or poor health, which then further contributes to more healthcare needs and subsequent environmental harm. GHGs advance climate change and negatively impact air quality, in turn negatively impacting health outcomes. As we continue to experience more frequent extremes including record heatwaves, our clinicians will continue to see its impact on respiratory, renal, and cardiovascular disease. Observational evidence suggests that heat plays a major role in deaths attributed to cardiovascular disease each year [11].

In the United States, the quest for sustainability started with the National Environmental Policy Act of 1969 which declared sustainability a national policy [12]. Since the enactment of the policy, there has been great interest among the public and stakeholders. The U.S. Environmental Protection Agency (EPA) publishes industry-specific reports including EPA/310-R-05-002 [13], which addresses the responsibilities and challenges of the healthcare industry. Such policies, if implemented well, may inform sustainability efforts that will likely produce a wide range of benefits for any organization, including financial (e.g., energy-saving) and operating efficiencies (e.g., waste reduction may result in reduced workload and streamlined processes), while supporting a growing green economy. However, implementing such policies may be challenging. Adding a layer of environmental sustainability considerations and logistics may appear to be a daunting task, especially in complex environments such as intenstive care units (ICUs).

The complexity of ICU operations may result in extensive waste generation compared to acute care units. For instance, a 12-bed intensive care unit in Brooklyn, New York, generated 7.1 kg of solid waste and 138kg carbon dioxide emissions per bed day [14]. The same hospital reported a 48-bed acute care unit generating 5.5kg of solid waste and 45kg of carbon dioxide emissions per hospital day [14]. Critical care has therefore been described as a locus of several of the healthcare industry’s “carbon hotspots” [15]. Despite the importance of sustainability for ICUs, the efforts in the United States have been limited and little has been done to summarize such efforts in this area to inform effective interventions. While the reason for such a paucity of sustainable healthcare initiatives is not well-documented, some (e.g., Richie, 2014 [16]) attribute this gap in most part to the political climate in the U.S. In this paper, we aim to review the current state of published sustainability efforts globally, specifically highlighting the U.S. critical care context; share our current sustainability efforts at a large, greater metropolitan area hospital system in Texas; and propose a pathway to a green ICU grounded in our exposure to various barriers and successes.

### Previous research on sustainability in critical care

Sustainability in critical care has been investigated in terms of various aspects including environmental, structural, and financial.

### Environmental approaches

Efforts have focused on quantifying carbon emissions and footprints [6, 9, 17, 18] to assess the impact of critical care on environment along the life cycle [8, 19–21]. Notably, Sherman et al. [17] proposed a comprehensive approach to sustainable healthcare emissions research based on a narrative review. This approach enables the top-down or bottom-up assessment of healthcare services as it frames the research studies around multiple levels including global supply chain, national healthcare sectors, healthcare systems, medical facilities (e.g., hospitals and clinics), clinical care pathways and procedures, and lastly individual drugs, medical devices, and basic materials. Yet, according to Huffling and Schenk [10], environmental sustainability in ICUs can be evaluated beyond carbon emissions, from the perspectives of waste (e.g., pharmaceuticals, medical or non-medical products and equipment), energy (e.g., lights, temperature settings, monitors, pumps, computers, TVs, batteries, and other equipment that seem to line the walls), toxic chemicals (e.g., air, dust, products, and food), and healing environment (e.g., noise, fast-paced tasks, and stress for staff as well as patients and their family members).

Waste in critical care has been of the topic of great interest to researchers and practitioners alike [17, 22–25], with various case studies published showcasing wasteful stocking and disposal practices. For example, Hunfeld et al. [21] reported that individual units used per ICU patient per day to be high as 108 disposable gloves, 57 compresses, 34 liquid medicine (infusion bags), 24 syringes, 23 tubes and connectors, 16 disposable clothing, 14 cups and containers, 11 tablets and capsules, 9 surgical masks, and 8 bed liners. The investigation of daily and best practices has centered on the actions of managing waste (without compromising safe and quality care) such as “reduce, reuse, recycle, and rethink” [15, 18, 19]. Those actions have been discussed alongside the incorporation of Lean Six Sigma—which emphasizes, among other process improvement techniques, continuous improvement in waste elimination [26]—and other quality management initiatives into critical care settings [25, 27]. A recent systematic review of waste management practices [25] found various types of interventions used in longitudinal studies including: policy changes, educational programs, operational procedure changes, waste sorting changes, Lean Six Sigma/total quality management, supply changes, and waste disposal changes. Notably, the COVID-19 pandemic has brought additional attention to waste management practices in critical care [24].

The waste management practices elicited from surveys, interviews, and observations of critical care professionals in Canada and Finland [27–29] have shown a common tendency to discuss barriers and facilitators to environmental sustainability based on patient care, organizational, and technological contexts. For instance, Kalogirou et al. [29] found that patient care and organizational contexts may physically and culturally influence the capabilities of professionals to promote and engage with responsible practices. Nurses participating in semi-structured interviews viewed environmentally sustainable practices to be at odds with both patient care priorities (e.g., patient care workload did not leave bandwidth to consider the environment) and with the organization’s priorities, support, and culture for strategic and operational management (e.g., when their organization puts budget as the top priority). On the other hand, Kallio et al. [28] and Yu and Baharmand [27] emphasized the utilization of functional facilities for waste sorting, training, and visible internal communications and reporting related to environmental sustainability.

### Structural and financial approaches

Structural and financial aspects of sustainability have also been investigated in critical care settings. Structurally, Halpern et al. [30] elaborated the evolution of ICU designs in the United States over four decades and highlighted that the evolution was guided by the shift from paper-based medical records to electronic health records. The technical shift has naturally required the support of advanced computers and displays, as well as other standalone informatics platforms such as physiological monitors, mechanical ventilators, infusion pumps, and beds. Financially, critical care has been characterized as expensive and wasteful; accordingly, sustainability efforts in critical care settings have been emphasized to decrease both healthcare costs and environmental hazards. For instance, Van Demark et al. [31] described their institutional efforts toward environmental sustainability in critical care with a project to reduce the amount of waste generated by hand surgery and showed decrease in both surgical costs and surgical waste while maintaining patient safety and satisfaction.

## Global trends in critical care sustainability

The World Health Organization (WHO) has emphasized the importance of sustainable healthcare practices globally, encouraging member states to develop and implement strategies that address environmental concerns. Accordingly, healthcare systems around the world have strived to integrate sustainability into ICU operations. Indeed, the intersection of sustainability and critical care (including surgical, medical, pediatric, and cardiac intensive care, burn care, and neonatal intensive care [13]) is well-studied with Europe [21, 28], the United Kingdom [8, 9, 32], Canada [22, 27, 29], and Australia and New Zealand [7, 33] at the forefront of such movement, including educational and advocacy materials [15, 19, 23, 34, 35]. European countries have made strides in adopting renewable energy sources and implementing energy-efficient technologies within healthcare facilities. In 2008, The European Union launched the Green Public Procurement (GPP), which is a process that guides sustainable purchasing decisions, including those related to ICU equipment and supplies [36]. Australia has led impactful initiatives such as the National Health Sustainability and Climate Unit [37] and National Health and Climate Strategy [38] which reflect a commitment to sustainability in healthcare, promoting energy efficiency, and responsible resource consumption in ICUs and other medical settings. Such initiatives have shown positive impacts on waste reduction. For example, an Australian staff-driven initiative reduced waste and increased recycling by replacing polystyrene beverage cups with recyclable cups and placing recycling stations in the ICU [39]. Recent evidence suggests that there is a dedicated clinician or team for Green initiatives in 65% of New Zealand ICUs and 40% of Australian ICUs as of the 2020–2021 financial year [40]. The Australian-based report *ANZICS: A Beginners Guide to Sustainability in the ICU* [33] and the resulting sustainability toolkit have been widely cited; however, this report and other prior work has generally not been widely translated into clinical impact, especially in the United States.

## Critical care sustainability in the United States

Broadly stated, there exists a significant gap in U.S. knowledge and published literature related to sustainability in the ICU [6, 10, 17, 20, 24, 25, 30, 31]. In addition, despite occasional features in society meetings, U.S. critical care societies have not released any position statements on the impact of sustainability in critical care. Indeed, sustainable ICU initiatives are in their infancy in the United States. In June 1998, the Hospitals for a Healthy Environment (H2E) was launched as a collaboration between the EPA and the American Hospital Association. H2E is currently a leading provider of tools and resources to help hospitals turn their operations green from front end materials purchased to back end waste management [41]. The group’s goals included total mercury waste reduction by 2005, overall hospital waste reduction of 33% by 2005 and 50% by 2010, and identifying additional substances to minimize/eliminate to prevent further pollution [42]. A follow up report was published by the EPA in May 2006 regarding the progress of these goals. It was noted that 75% of H2E partners had completely eliminated mercury-containing devices and 90% of hospitals had reduced mercury-containing devices [43]. However, at the time of writing, no progress has been made on the waste and pollution reduction initiatives.

In 2009, the U.S. Green Building Council (USGBC) created the Leadership in Energy and Environmental Design (LEED®) reference guides and rating systems for building design, construction, and existing operations (as amended and expanded to include healthcare) [44–46]. This guide is a toolkit and rating system for sustainable design and operations in healthcare facilities, including critical care areas. It contains recommendations such as the implementation of energy-efficient technologies, such as LED lighting and high-efficiency HVAC systems, contributing to reduced energy consumption and operational costs [46]. Complementing the LEED for Healthcare rating system as a third-party form of certification, the *Green Guide for Health Care*™ (GGHC) is a voluntary self-certifying tool and joint project of Health Care Without Harm and the Center for Maximum Potential Building Systems; GGHC represents a culmination of several years of close collaboration with and guidance from the USGBC [47, 48].

LEED for Healthcare was written primarily for inpatient and outpatient care facilities and licensed long-term care facilities. It can also be used for medical offices, assisted living facilities, and medical education and research centers. LEED for Healthcare addresses design and construction activities for both new buildings and major renovations of existing buildings. For a major renovation of an existing building, LEED for Healthcare is the appropriate rating system. If the project focuses more on operations and maintenance activities LEED for Existing Buildings: Operations and Maintenance is more appropriate [45, 46].

Several awards and distinctions have been established to recognize hospital systems for their efforts in sustainability in the healthcare realm. Practice Greenhealth is an organization that focuses on sustainability solutions for healthcare systems. In 2023, they named 25 hospitals to receive the Environmental Excellence Award for hospitals leading in healthcare sustainability performance [49]. The Greenhealth Emerald Award is an honor given to the top 20% of Partner for Change applicants and recognizes hospitals that have excellent sustainability programs and superior scores in multiple sustainability categories [50]. Additionally, Becker’s Healthcare has published a list several years running of the “Greenest Hospitals in America,” selected based on nominations and editorial research [51]. These honors provide a benchmark for hospital and healthcare systems to strive for when creating their sustainability programs.

### Our sustainability initiative

Houston Methodist (HM) is establishing a firm commitment to creating an environmentally sustainable healthcare institution. HM is a health system comprising eight hospitals throughout the Greater Houston metropolitan area (a 13-county region spanning over 10,000 square miles with a demographically diverse epicenter [52]) including Houston Methodist Hospital, the flagship academic hospital in the Texas Medical Center, and six community hospitals, as well as one long-term acute care hospital and a seventh community hospital under construction (as of writing).

Unit- and department-specific sustainability efforts depend on broader organizational support and prioritization [29]. Earlier this year, HM established an Office of Sustainability to oversee and direct the responsible use of resources to conserve the environment and to support system-wide efforts that balance economic viability, social equity, and environmental protection. HM has already rolled out important environmental sustainability initiatives. For example, the system is currently in the design phase for installing solar panels on some of its main buildings in the Texas Medical Center. This project, in partnership with Houston Methodist's Energy and Facilities workgroup, will be the first step toward renewable energy consumption for the hospital. HM has also launched food composting initiatives at its community hospital locations in Sugar Land, The Woodlands, and Willowbrook—with plans for additional campuses to follow. According to the Office of Sustainability, the hospital system has already diverted nearly 100,000 lbs. of food waste from landfills. HM also focuses on preventing waste by recycling or reusing items, from creating a workflow that enables reusing items that can be sanitized to sustainably disposing of expired materials. Finally, another notable initiative is incorporating greenspace for patients to enjoy. Houston Methodist Hospital is currently constructing a 26-story hospital tower that will feature the Centennial Rooftop Garden on the 14th floor.

Several ICU-based projects are in various stages of implementation throughout our hospital system. Most of these initiatives have a low barrier to entry with minimal need for additional personnel or equipment and therefore negligible cost implications. For instance, multiple ICU units within our health system are examining strategies to reduce the amount of unused supply waste, an identified priority considering just one of the ICUs in our health system was found to use 1,464,262 medical supplies in a 6-month period. Some interventions include staff education, changing the supplies in premade procedure kits, using a procedure cart to store supplies, and creating an airway box for intubation supplies. Staff education includes providing awareness of the issue of bringing a surplus of supplies into a patient’s room as well as inappropriately opening the code cart for a supply that is available in another area within the ICU. In addition to reducing excess supplies, teams are attempting to reduce the amount of unnecessary oxygen used in the ICU.

To meet escalating critical care needs, HM also launched a systemwide virtual ICU (vICU) program [53, 54], with potential low-carbon implications. This state-of the-art facility leverages the digital transformation of in-hospital care to incorporate remote monitoring and interactive video conferencing. The vICUs’ “consultant bridge” application allows virtual specialist patient consultations, virtual family visits, tele-rounding, and reduction in staff commuting—innovations that reduce travel-associated carbon without compromising the quality and safety of patient care [55]. These contributions to reducing the carbon footprint are expected to be significant considering that they target critical care’s specific “carbon hotspots” in the healthcare sector [15], and that similarly significant carbon footprint reductions have been observed for telemedicine programs in broader healthcare delivery contexts in both the U.S. and internationally [56–59]. In our tele-critical care experience, and in line with other telemedicine reviews [60, 61], telemedicine results in the reduction of interhospital transfers, enabling remote patient evaluations that decreases unnecessary patient transports to tertiary care centers, resulting in potentially singificant cut in the carbon footprint associated with such long-distance travel. It should be noted, however, that studies do not consistently consider additional factors beyond travel in the the emission calculations [56], e.g., energy and equipment requirements for virtual hubs may require further study.

While the initial programs may seem simplistic in nature, we anticipate barriers to arise as the initiatives expand. These barriers include buy-in from the stakeholders involved in the interventions, resistance to change, and longevity of programs given the nature of human behavior to revert to old habits. In addition, as inititives become more resource-intensive (such as the installation of solar panels), we anticipate even more resistance and significant financial and administrative barriers. Finally, collecting pre- and post-intervention data may impose new workflows, added to already high workloads, and require additional resources. These anticipated barriers underscore the need for continuous stakeholder engagement to inform developments.

### A proposed pathway for sustainable ICUs

Grounded in our experience with HM Green ICU efforts, we propose a 3-step pathway to inform similar initiatives for sustainable (green) critical care. The first step in creating an environmentally sustainable ICU is to establish a baseline by quantifying the status quo carbon footprint of the affected ICU as well as the cumulative footprint of all the ICUs in the healthcare system—a step that will require collaboration and partnership with different departments and stakeholders across the system; sustainability effort is a team commitment where each stakeholder, including the clinician leaders and not just administrators and operational leadership, needs to be aware and involved. ICUs and acute care facilities contribute significantly to a hospital’s overall GHG emissions and its solid waste generation. The second step is to form alliances and partnerships to target each major source of these pollutants and implement specific intervention programs that reduce the ICU-related GHG emissions and solid waste. In the third step, successful implementation of a systemwide Green ICU will require the creation of multiple parallel pathways that marshal the resources at the grass-roots level to engage the ICU staff and institutionalize a mindset that recognizes and respects the impact of ICU functions on our environment. These steps are detailed below.

### Step 1: Conduct life cycle assessment of ICU products and processes

To establish a baseline for ICU carbon footprint, environmental experts should be engaged to carry a comprehensive audit of the ICU, quantifying the financial and nonfinancial cost of all the inputs and outputs of ICU operations. For example, sustainability teams or offices may partner with local universities that have an established environmental sustainability program to carry out such an audit. Table 1 summarizes some of the proposed audit components.

**Table 1:** Summary of proposed ICU carbon footprint audit components and their associated inputs and outputs

Selective Components	Inputs	Outputs
Building utilities and infrastructure (e.g., heating, ventilation and air-conditioning [HVAC] system, water, and electricity)	Natural gas Water treatment Fuel processing Generation Mix (coal/renewables)	Electricity usage per patient/bed Waste water generation
Manufacturing pharmaceuticals, linens, and ICU equipment, instruments & supplies, including medical gases (oxygen, nitrous-oxide), portable ultrasound machines, defibrillators, medical dispensers, warming units, laryngoscopes, laryngeal mask airways, and dental burs	Raw materials Energy Utilities Transportation Labor Manufacturing processes	Pharmaceutical waste Laundry Sharps/red bag waste Water and air pollution
Nutrition (e.g., patient meals)	Produce Agricultural Infrastructure & food delivery logistics	White bag waste Recyclable waste

This audit should provide GHG emissions and solid waste generation per patient, ICU bed, and square footage of the physical space. Another component that may be included is the impact of transportation of ICU staff and patients from home or other medical facilities to the ICU. Medical transport, such as “life flight” services may have significant impact on the environment worth quantifying in future studies. In addition to establishing the status quo of the ICU carbon footprint, the audit teams should provide guidelines about what level of reduction in the carbon footprint would be pragmatic and achievable over a clearly defined period.

When looking to determine emissions and utilize emissions factors, many companies offer software suites that healthcare systems may find costly. While we hope to see lower cost/no cost access to user friendly emissions quantification systems, other resources are available for less resourced settings. When looking to quantify emissions, emissions factors are available through the U.S. Environmental Protection Agency [62] and Greenhouse Gas Protocol website of the World Resources Institute and World Business Council for Sustainable Development [63]. Additionally, Practice Greenhealth and Health Care Without Harm provide resources or direction to resources, including the GGHC as discussed above.

### Step 2: Develop green ICU interventions through strategic partnerships

Most health systems and hospitals have formed an office or executive role for sustainability, or minimally may have executives willing to champion sustainability efforts. Partnerships with such offices will allow collaboration with many stakeholders and decision makers outside of the ICU walls to create intervention “bundles” inside and outside the ICU to reduce the carbon footprint by a defined target amount over a stated period. Table 2 summarizes several areas that may be addressed by the experts from outside the ICU such as facilities management and IT.

**Table 2:** Proposed areas for external consultation related to sustainability initiatives

Area for external input	Description
Energy conservation	Heating, ventilation, and air-conditioning (HVAC) systems within a hospital and an ICU are generally very energy-intensive and contribute significantly to the GHG emissions. Changing incandescent to fluorescent and LED lighting should help in reducing the GHG emissions and saving costs over the long term. Deploying occupancy sensors in all areas other than patient care and medication preparation areas, would ensure that lights are turned off automatically when not in use
Temperature setting	Heating and cooling systems should be centrally set at optimal temperature for various parts of the building, while allowing manual control over sensitive areas such as operating rooms, labs, and data centers
Computers and TV	The IT department should manage the computer monitors and TVs using a power management system that puts these devices in hibernation if left idle for an extended period
ICU windows	ICUs with windows that face out should be equipped with blinds or other shades that insulate from the heat during the summer and allow the warm sunlight to enter during the cold months
Energy efficient procurement	Wherever possible, offices of sustainability should procure capital equipment and supplies for the ICUs from manufacturers who have demonstrated a commitment to using energy efficient technologies and raw materials. Procurement practices should reward with greater share of the business those manufacturers who have made structural adjustments to containers and packaging of clinical supplies to reduce the amount of plastic/resin being used
Green spaces	To offset the pollutants created by ICU operations, hospitals may create new or designate existing physical space as “green space” with the goal of net zero GHG emissions. While this is ideal, real estate is a scarce resource for most urban hospital settings and operationalization of such green spaces may be challenging. When considering a new build or retro-commission, mass timber offers a light, affordable, and sturdy building material option embodying carbon that it has sequestered during its lifetime

### Step 3: Create green ICU teams comprised of ICU staff—*a grass-roots effort*

To create a culture and mindset of “green ICU” with the overarching goal of mitigating the ICU-generated pollutants, several Green Teams should be formed, each tasked with implementing a “bundle” of environmental interventions. The Green Teams should include representation by all the functional roles of the facility, including doctors, nurses, technicians, administrators, and custodial staff.

Green Teams serve as the local champions for sustainability and can take the lead in creating the culture for “green” thinking. Guidelines focusing on 3Rs (“Reduce / Recycle / Rethink”) along with such strategies as “Less is More” should be used as educational tools to increase awareness of the impact of resource usage in the ICU. The guidelines, however, should give priority to the demands of patient care. No environmental sustainability directive should compromise the heath and safety of patients or override the judgement of physicians and family members. Table 3 summarizes some of the areas that may be addressed by the 3R team structure.

**Table 3:** A proposed 3Rs green team structure with proposed areas of responsibility

Green team	Areas of reponsibility
Recycle team	Introduction of recycling stations within the ICU Encourage accurate waste segregation Recyclable materials: Paper, plastics, glass, metals Used fluid bags and non-sharps
Reduce team	Guidelines for reducing unnecessary use of supplies and equipment Gloves, linens, gowns Non-critical drugs (oral/IV) Reduce consumable waste Trolley supplies Streamline prefabricated kits Switch from single-use supplies to reusable ones Reduce use of energy by utilizing sunlight, better ventilation, water conservation Repurpose, repair, and refurbish, whenever possible
Rethink team	Review all the protocols to minimize waste and pollution Develop new processes and policies to empower recycle and reduce efforts Educate the staff and other stakeholders Liaise with senior management to coordinate institution-wide environmental sustainability initiatives

#### Recycle team

Certain products can significantly reduce pollution from medical waste. The goal for recycling is reduction of landfill waste and reduction of costs for facilities that purchase the recycled items. Difficulty in sorting the plastic waste and risk of transmitting potential infections limit the practice of recycling medical supplies. Placement of recycling containers for adequate sorting of products is essential. These items can be sent to a third-party facility where items are cleaned, sterilized, and sold back to hospitals for discounted rates [14, 64, 65].

#### Reduce team

One of the main responsibilities of the reduce team is to identify opportunities for conservation. The complexity of critical care requires large quantities of medical supplies needed for patient care. Infection control precautions demand single use packaging which creates high frequency of plastics waste. Nursing staff often anticipate the use of supplies and pre-stock the room, which results in items that go unused and unopened. This practice creates a surplus of medical supply waste, specifically in the isolation rooms. It is important to raise awareness over infection control policy regarding restrictions of medical supplies taken into isolation rooms. Understanding that unopened supplies will be discarded may trigger staff to help conserve medical supplies. Another option to raise awareness about conservation practices could be to make a price list of supplies. Cognizance over the monetary cost of supply waste may trigger staff to conserve.

#### Rethink team

Improving awareness by providing education about the recycle and reduce efforts may play a major role in increasing readiness for change to accommodate new policies and processes related to sustainability. For example, education on the composition of recyclable items will be an important part of the green initiative. Plastic recycling can be categorized by ease of recycling type. Education over categories of plastic may help staff understand which supplies are recyclable.

**Fig. 1:**
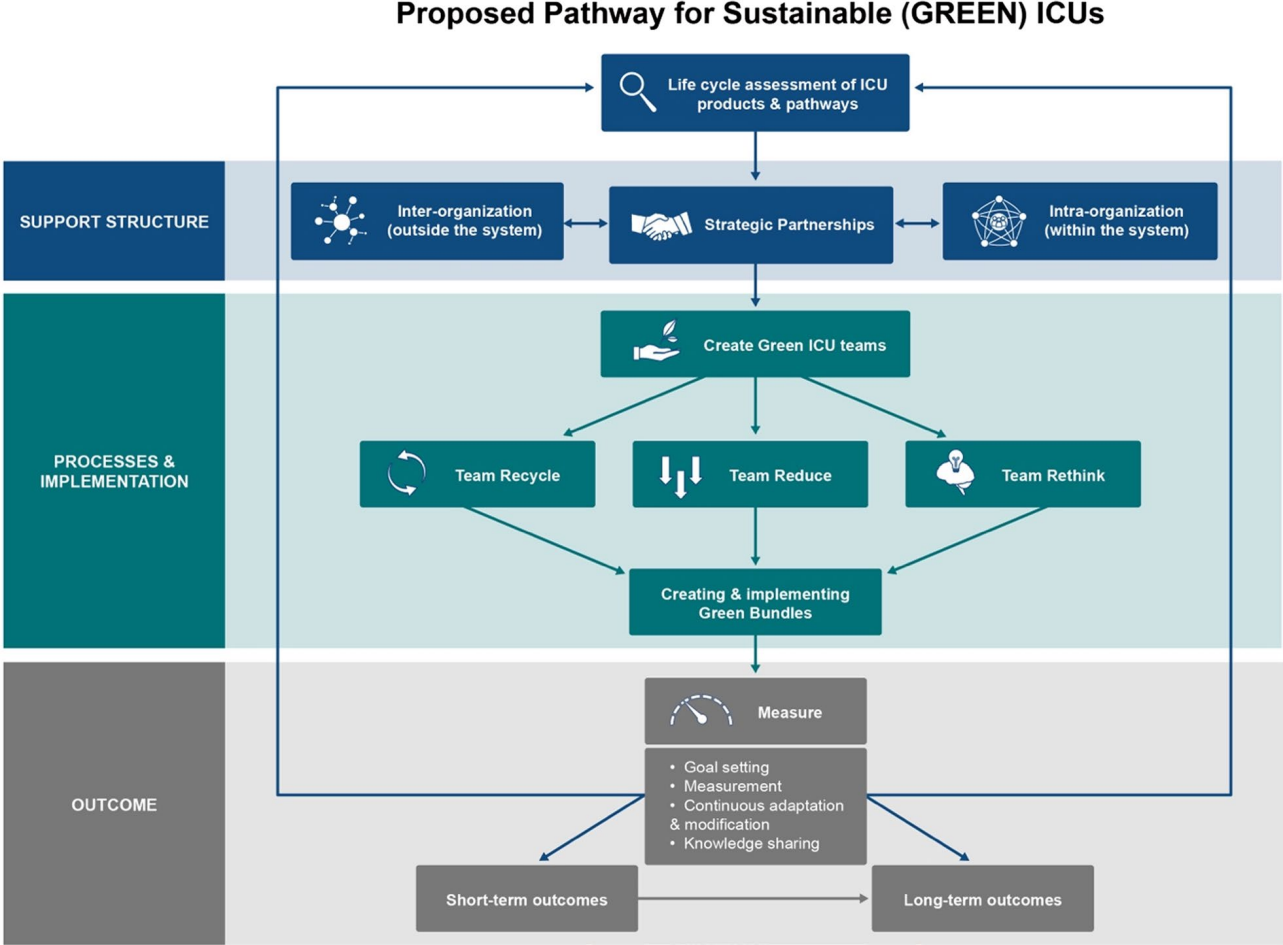
Overview of the porposed pathway for sustainable (Green) ICUs

Once the environmental sustainability guidelines have been established for the ICU and the intervention programs have been implemented, periodic progress checks would be needed to measure the effectiveness of the program and possible impact on the ICU footprint. It must be emphasized that the environmental sustainability programs should not compromise quality of patient care and patient safety. For example, switching from single-use to reusable equipment should not increase the risk of infection for the ICU patient. Figure 1 provides an overview of the proposed pathway.

## Conclusions

While the precise accumulated negative environmental effect of thousands of ICUs across the United States remains unknown, such effects represent a significant portion of the healthcare industry’s contribution to the overall carbon footprint. While efforts are in place to improve sustainability in ICUs, there is a general gap in implementation of effective interventions globally and especially in the United States. This paper presents a pathway for such intitiaves grounded in our implementation of a Green ICU in a large health system. A systems approach that involves various stakeholders is necessary to create a plan for effective recycling of medical supplies, reducing unnecessary supplies, and raising awareness of the urgency and value of such initiatives. The proposed pathway has minimal requirements for additional resources and is expected to generalize to a wide range of health systems with varying levels of resources.

## Data Availability

Not applicable.

## Abbreviations

EPA: United States Environmental Protection Agency
GGHC: Green Guide for Health Care
GHG: Greenhouse gas
GPP: Green Public Procurement
H2E: Hospitals for a Healthy Environment
HM: Houston Methodist (a Greater Houston area hospital system)
ICU: Intensive care unit
LEED: Leadership in Energy and Environmental Design
USGBC: United States Green Building Council
